# Upregulation of the *ERG11* gene in *Candida krusei* by azoles

**Published:** 2010

**Authors:** M. Tavakoli, F. Zaini, M. Kordbacheh, M. Safara, R. Raoofian, M. Heidari

**Affiliations:** 1 Department of Parasitology and Mycology, School of Public Health; 2 Department of Medical Genetics, School of Medicine, Tehran University of Medical Sciences, Tehran, Iran

**Keywords:** Polymorphism, Drug resistance, Gene expression, RT-PCR

## Abstract

**Background and the purpose of the study:**

*Candida* species are the agents of local and systemic opportunistic infections and have become a major cause of morbidity and mortality in the last few decades. Azole resistance in *Candida krusei* (*C. krusei*) species appears to be the result of gene alterations in relation to the ergosterol biosynthesis pathway, as well as efflux pumps. The main objective of this study was to examine the RNA expression of *ERG11* in *C. krusei* which had been identified to be resistance to azoles.

**Methods:**

The *ERG11* mRNA expression was investigated in four Iranian clinical isolates of *C. krusei,* which were resistant to fluconazole and itraconazole by a semiquantitative RT-PCR. *Results*: The mRNA expression levels were observed in all four isolates by this technique. Furthermore, it was found that *ERG11* expression levels vary among four representative isolates of *C. krusei*. Although DNA sequencing revealed no significant genetic alteration in the *ERG11* gene, one heterozygous polymorphism was observed in two isolates, but not in others. This polymorphism was found in the third base of codon 313 for Thr (ACT>ACC).

**Major conclusion:**

Even though such a polymorphism creates a new *Ear1* restriction site, no significant effect was found on the resistance of *C. krusei* to azoles. Results of this investigation are consistent with previous studies and may provide further evidence for the genetic heterogeneity and complexity of the ergosterol biosynthetic pathway or efflux pumps.

## INTRODUCTION

*Candida* species (spp) are agents of local and systemic opportunistic infections worldwide and have been described as the fourth leading cause of nosocomial bloodstream infections (BSIs). Moreover, treatment failures and development of the drug resistance have frequently been reported (1). Although C. *albicans* is the most important cause of candidemia, an increasing number of infections due to non *Candida albicans* species such as *C. glabrata* and *C. krusei* have also been reported (2). On the basis of reports 95% of *Candida* BSIs are associated with *C. albicans, C. parapsilosis, C. glabrata,* and *C. tropicalis* species and 12–14 of other *Candida* spp are involved in 5% of BSIs (3–5). A slight increase of BSIs due to non*-albicans* species has been reported, and *C. krusei* accounts for 24% of all *Candida* nosocomial bloodstream infections. It is known that this species has a tendency to appear in a setting where fluconazole has been administered for prophylaxis (5). Colonization and infection with fluconazole-resistant *Candida* spp. has often been observed among high risk patients with hematological malignancies under the selective routine fluconazole prophylaxis (6, 7). *C. krusei* has been detected as an uncommon and potentially multi-drug resistant (MDR) pathogen. In vitro antifungal testing has shown a considerable reduction in susceptibility of *C. krusei* to fluconazole (2.9% sensitive) and amphotriecin B (8% of all isolates), and the emerging pathogenicity of this organism is of increasing public health concern (8). It has been demonstrated that multiple mechanisms are involved in azole resistance, including overexpression of several genes encoding efflux pumps such as CDR1, CDR2 and MDR1 (multi-drug resistance), which lead to reduced intracellular accumulation of fluconazole and overexpression of the *ERG11* gene, coding for the sterol 14α-demethylase (9, 10).

Some studies have proposed reduction in susceptibility of sterol 14α-demethylase to fluconazole as major resistance mechanism in *C. krusei* (11–14). The present study was aimed to investigate the expression of *ERG11* in four Iranian *C. krusei* isolates.

## MATERIAL AND METHODS

### 

#### Fungal strains

Four fluconazole and itraconazole resistant *C. krusei* strains were included in the present study (Table 1). These strains were isolated from cancerous patients with oropharyngeal *Candida* infections during 2006–2008 and had been identified previously (15). The susceptibility testing of the isolates to fluconazole and itraconazole was performed according to the National Committee for Clinical Laboratory Standards M27-A (NCCLS) by broth microdilution method (16). Susceptibility tests were carried out in RPMI 1640 medium (SigmaAldrich, USA) buffered to pH 7.0 with 0.165 M morpholinepropanesulfonic acid (MOPS). The MICs were defined as concentrations of the drug that reduced growth by 80% compared to that of organisms grown in the absence of the drug. NCCLS-recommended quality control (*Candida krusei* ATCC 6258) was included in each test run, and MICs were within the recommended range for each test. The isolates had been stored in glycerol/ water at −80°C until used. The inocula for each individual experiment were prepared from these stocks.

**Table 1:** Characteristics of *Candida krusei* strains used in this study.

Strain	Predisposing factor	Site of isolation	MIC^a^ (ng/ml)	

			FLC^b^	ITC^c^
*C. krusei 2*	Carcinoma (lung)	Orophrynex	64	1
*C. krusei 118*	Lymphoma	“	128	1
*C. krusei 124*	Lymphoma	“	64	2
*C. krusei 144*	Leukaemia	“	64	2

a : Minimum Inhibitory Concentration

b :Fluconazole

a :Itraconazole

#### Total RNA and cDNA synthesis extraction

*C. krusei* cells were grown on Sabouraud dextrose agar at 37°C for 24 hrs. Two to three fresh colonies were transferred to yeast peptone dextrose (YPD) broth (yeast extract 1%, peptone 2%, dextrose 2%), (Suprapur, Merck, Germany) at the same temperature for 48 hrs.

Total RNA was isolated from exponential-phase of the YPD broth cultures using RNeasy Mini kit (Qiagen) according to the manufacturer's instruction. Quantification of RNA was performed by absorbance at 260 nm using a Spectrophotometer (Biophotometer). The mean RNA concentration and the mean ratio for OD260/280 were 421+6 ng/µl and 1.8+0.04, respectively. For cDNA synthesis, 10 µl of total RNA was heated in 80°C for 10 min followed by cooling on ice. The master mixture contained 4 µl of 5x reverse transcriptase (RT) buffer, 10 mM of each dNTP, 20 pmol/µl random primer, 20 U RNase inhibitor (Fermentas, Burlington Canada), 200 U of Moloney Murine Leukemia Virus (MMuLV) Reverse Transcriptase (Fermentas, Burlington Canada), and 1.5 µl of DEPC-treated water. The cDNA synthesis was performed under following conditions: 42°C for 60 min, 70°C for 10 min, and finally cooling to 4°C. The integrity of cDNA was checked using the house keeping gene 18sRNA primers (as shown in table 2) which amplify region 1433–1639 (GB. EU348783.1). Samples with similar cDNA quality through 18sRNA PCR were stored at -70°C for further investigation.

**Table 2:** List of primers used in this study.

Primer Name	Primer Sequences 5′→3′	Accession Number	PCR Product Sizes (bp)
Semi-Quantitative RT-PCR Primers			

18SCF	5′-GACGGAGCCAGCGAGTATAA-3′	GB.EU348783.1	206
18SCR	5′-GGGCTCACTAAGCCATTCAA-3′		

*ERG11F*	5′-AATGGGTGGTCAACATACTT-3′	DQ903905	508
*ERG11R*	5′-TGGTGGTAGACATAGATGTATT-3′		

Sequencing Primers			

ER11SF	5′-GTTTACGGAAAACCTTAC-3′	DQ903905	1218
ER11SR	5′-GGTACATCTATGTCTACCACCACCA-3′		

PCR amplification of the *ERG11* gene was conducted on samples using 1 µl of cDNA, specific forward and reverse primers corresponding to *ERG11*gene (Table 2), dNTP, MgCl, Taq DNA polymerase, and buffer (CinnaGen, Tehran, Iran). The thermocycling was performed using a Touch-Down amplification program on 2720 Thermal Cycler, ABI. The PCR condition was as the same as previously described (17).

#### Semi-quantitative RT-PCR

Semi-quantitative RT-PCR was conducted by the reported method with minor modification (18, 19). Briefly, specific primers corresponding to *ERG11* and 18s rRNA mRNA sequences were designed. Appropriate dilutions of the samples were determined for each cDNA to make sure that examined transcripts and 18sRNA (internal control) amplification was in the exponential phase of the reaction.

#### DNA sequencing

For mutation screening, genomic DNA was extracted from 5×107 cells using DNGPLUS kit (CinnaGen, Tehran, Iran). PCR amplification of whole *ERG11* gene was carried out using specific primer pairs (Table 2). To detect any mutation, the PCR product was subjected to direct sequencing (Gen-Fanavaran, Tehran, Iran). Sequence data searches were performed in non-redundant nucleic and protein databases BLAST (http://www.ncbi.nlm.nih.gov/ BLAST).

## RESULTS

**Figure 1:**
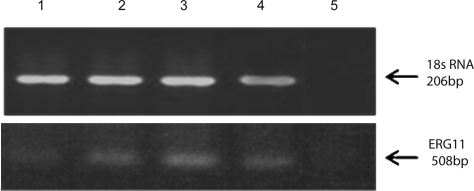
*ERG11* mRNA expression. RT-PCR products of *ERG11* gene of clinical isolates of *C. krusei*, resistant to fluconazole and itraconazole on 1.8% agarose gel stained with ethidium bromide. Lanes 1(+1), 2 (+4), 3 (+3) and 4 (+2): indicate different levels of *ERG11* mRNA expression (508bp); lane 5: negative control (water).18srRNA (206bp) was used as a positive control.

Four *C. krusei* isolates exhibited *ERG11* mRNA overexpression at various levels. A semiquantitative RT-PCR was used to compare positive results of expression levels as: no expression (0), mild (1+), moderate (2+), high expression (3+) and the highest expression (+4) (Fig. 1).

**Figure 2:**
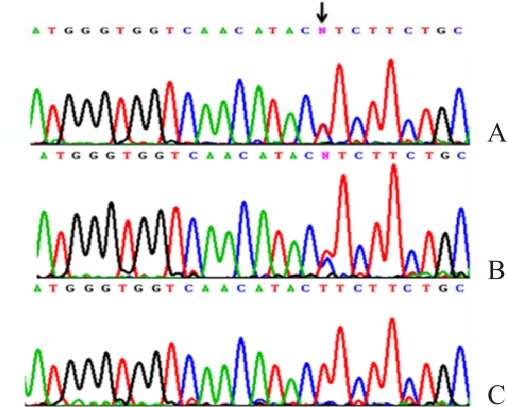
Mutation analysis of the ERG11 genomic DNA. (A, B) A heterozygous polymorphism in codon 313 for Thr (ACT>ACC) in which T→C at position 939 of mRNA (DQ903905) was found in two samples. (C) A chromatogram from one of the samples indicates wild type homozygous condition.

Direct DNA sequencing was carried out for investigation of the molecular bases of ERG11 overexpression. Amplified PCR products of the complete coding sequences of this gene from four *C. krusei* isolates were sequenced. The chromatogram of *ERG11* DNA sequencing (Figures 2A, B) indicated a heterozygous base-substitution in two *C. krusei* isolates, in which a heterozygous change had occurred in the third base of codon 313 for Thr (DQ903905). Figure 2 C shows homozygous condition of the mentioned polymorphism. Although this genetic alteration (ACT>ACC) can not change the amino acid sequence of the *ERG11* protein, it leads to the creation of an *Ear1* restriction enzyme recognition site. Also, direct PCR sequencing of other samples revealed no mutation (data not shown).

## DISCUSSION

Ergosterol biosynthesis is a complex metabolic pathway. So far, the involvement of several genes encoding enzymes in this pathway has been identified. It has been well documented that some of these metabolic steps are critical for cell viability. For instance, *ERG11* deletions are lethal in *S. cerevisiae* (9), whereas no specific gene in *C. krusei* has been reported to exert significant effect. Previous studies have shown the role of *ERG11* upregulation in fluconazole-resistant clinical isolates of *Candida* spp. (20). Several lines of evidence suggested that other genes of the sterol biosynthetic pathway (*ERG3*) and another pathway such as efflux pumps also play critical roles in the antifungal resistance of yeast (21–24). Moreover, some studies have shown involvement of efflux pumps in increasing the levels of resistance of *C. krusei* to azoles (25–30). In this study a semi-quantitative RT-PCR employing 18sRNA as an internal control was performed for detection of the levels of *ERG11* expression in clinical isolates of *C. krusei*. All isolates revealed variations in *ERG11* expression levels. To date, more than 20 genes have been found that are involved in azole resistance.

In general, *ERG* genes were found to be unregulated by either the reduction of a late product; or an accumulation of an early substrate or toxic sterol intermediates of the ergostrol biosynthetic pathway. It has been clearly demonstrated that several different inhibitors can affect different enzymes of this pathway which lead to upregulation of *ERG* genes in *S. cerevisiae* and *Candida* spp. Moreover, most previous studies have shown that the levels of ergosterol or other intermediate sterols which are formed in this pathway might be responsible for regulation of *ERG* expression in these species (20, 30). The exact molecular mechanism behind the upregulation of *ERG11* gene in response to azoles and other antifungal drugs is not completely understood. Therefore, it was considered to investigate whether various amino acid substitutions or probable mutations, cause enhanced expression of *ERG11* and changes in azole susceptibility (31). Our DNA sequence analysis of the *ERG11* coding region displayed a heterozygous base substitution T→C (ACT>ACC) in two *C. krusei* isolates. However, this genetic alteration cannot lead to a change in the amino acid sequence of *ERG11* protein which creates an *Ear1* restriction enzyme recognition site. It has been well documented that protein- DNA interactions play a fundamental role in cell biology (32). Therefore, this polymorphism might play a critical role in the transcriptional regulation of genes which might be involved in the processes of ergosterol biosynthesis.

In addition, data of this study revealed that *C. krusei* is a diploid organism, which is in agreement with most recent findings which have identified different alleles for *ERG11* gene in *C. kruse* strains (27).

## CONCLUSION

The *ERG11* overexpression is unlikely to be the cause of azole resistance in *C. krusei* isolates. A potential mechanism for azole resistance could be the level of promoter activity which is equivalent to the rate of ergosterol or sterol biosynthesis, as it has been confirmed in yeasts and human beings. In fact, it appears that stimulation of the *ERG11* promoter has arisen in response to sterol deprivation or a reduced level of sterol resources. It is noteworthy that upregulation of *ERG11* could be affected by its upstream (*ERG9, ERG1, ERG7*) or downstream genes (*ERG3*, *ERG25*). Additionally, genetic changes in the *ERG11* promoter region may also modulate levels of the expression of genes that are involved in ergosterol biosynthesis. Other possibilities to explain *ERG11* overexpression and azole resistance might be due to the growth conditions, carbon source and semi-anaerobic growth of fungal cells.
